# Nationwide Serological Survey of Equine Trypanosomosis in Kazakhstan

**DOI:** 10.3390/pathogens15030303

**Published:** 2026-03-11

**Authors:** Ainur Nurpeisova, Zhadra Kudaibergenova, Roza Aitlessova, Bolat Shalabayev, Maksat Serikov, Altynai Arysbekova, Makay Zheney, Nuray Ibraim, Kobeikhan Begassyl, Rano Sattarova, Kuandyk Shynybayev, Raikhan Nissanova, Indira Akzhunusova, Nurkuisa Rametov, Zhibek Zhetpisbay, Han Sang Yoo, Nurlan Ahkmetsadykov, Kunsulu Zakarya, Markhabat Kassenov, Zhandos Abay

**Affiliations:** 1 Kazakh Scientific Research Veterinary Institute LLP, Almaty 050016, Kazakhstan; nurai1005@gmail.com (A.N.); zhadra.n.k@gmail.com (Z.K.); rozaajtlesova@gmail.com (R.A.); bolat.shalabayev@gmail.com (B.S.); kazpatent@bk.ru (M.S.); arysbekovaaltynaj@gmail.com (A.A.); j.magay_06.10@mail.ru (M.Z.); nurai.ibraim08@gmail.com (N.I.); thekobi2014@gmail.com (K.B.); ranosaitomarovna@gmail.com (R.S.); shynybaev.k@gmail.com (K.S.); raihan.nisanova@gmail.com (R.N.); indiraakzunusova@gmail.com (I.A.); kasenovmarhabat@gmail.com (M.K.); 2 Faculty of Veterinary Medicine and Animal Science, Kazakh National Agrarian Research University NJSC, Almaty 050010, Kazakhstan; nurlan.akhmetsadykov@gmail.com; 3 Tecton Analytics LLP, Astana 010000, Kazakhstan; nurkuisa.rametov@gmail.com; 4 Department of Computer Science, Al-Farabi Kazakh National University, Almaty 050040, Kazakhstan; zhibekzhetpisbay@gmail.com; 5 College of Veterinary Medicine, Seoul National University, Seoul 08826, Republic of Korea; yoohs@snu.ac.kr; 6 Scientific and Production Enterprise “Antigen” LLP, Almaty Region 040905, Kazakhstan; 7 National Holding “Qazbiopharm” JSC, Astana 010000, Kazakhstan; krzakarya@gmail.com

**Keywords:** dourine, *Trypanosoma equiperdum*, equine trypanosomosis, seroprevalence, complement fixation test

## Abstract

Equine trypanosomosis remains an important veterinary concern in regions where horses play a significant economic and cultural role. In Kazakhstan, comprehensive nationwide data on the seroepidemiological status of equine trypanosomes are limited. The aim of this study was to assess the serological distribution of equine trypanosomosis across all administrative regions of Kazakhstan using complement fixation testing (CFT). A total of 6065 equine serum samples were collected from seventeen regions between 2023 and 2025. Antibodies against members of the *Trypanozoon* subgenus were detected using a WOAH-recommended CFT protocol. Overall seropositivity was 4.73%, with substantial regional variation ranging from 0% to 16.52%. Statistically significant differences in seroprevalence were observed between regions (*p* < 0.001), and mixed-effects modelling indicated considerable regional clustering. PCR testing of seropositive samples did not confirm the presence of *Trypanosoma equiperdum*, while one sample tested positive for *Trypanosoma evansi*. These findings suggest that CFT seropositivity reflects exposure to equine trypanosomes rather than confirmed dourine infection. Given the inability of CFT to reliably distinguish between *T. equiperdum* and *T. evansi*, species-level attribution remains uncertain. This study provides the first nationwide overview of serological reactivity to equine trypanosomes in Kazakhstan. The results highlight regional heterogeneity in antibody detection and underscore the need for expanded molecular surveillance and improved species-specific diagnostic tools to clarify the epidemiological status of equine trypanosomosis in the country.

## 1. Introduction

Animal trypanosomiasis comprises a group of protozoan diseases caused by parasites of the genus *Trypanosoma*, several of which are of veterinary and zoonotic importance [1,2,3,4]. Among these, *Trypanosoma equiperdum* (*T. equiperdum*), the causative agent of dourine, represents a particular concern for equine health due to its chronic course, significant economic impact, and diagnostic challenges [5,6].

Previous studies have suggested that some isolates historically classified as *T. equiperdum* may in fact represent misidentified strains of *Trypanosoma evansi* (*T. evansi*), and that both *T. evansi* and *T. equiperdum* evolved independently from *Trypanosoma brucei* [7,8]. Dourine is a chronic and often debilitating disease of equids that primarily affects horses and poses a serious threat to breeding programs and animal trade [9]. Unlike other pathogenic trypanosomes, *T. equiperdum* is transmitted almost exclusively through sexual contact between equids, without the involvement of an obligatory invertebrate vector [9,10]. The parasite predominantly localizes in tissues, particularly within the genital tract, and is rarely detectable in peripheral blood, which complicates direct parasitological and molecular diagnosis [11,12,13]. Consequently, infected horses may remain asymptomatic for long periods while continuing to act as sources of infection.

In Kazakhstan, equine trypanosomosis is of particular relevance due to the country’s large horse population and the active national and international movement of breeding animals. Two trypanosome species are regarded as veterinary pathogens in the region: *T. equiperdum*, responsible for dourine in horses, and *T. evansi*, the causative agent of surra, which primarily affects camels but can also infect equids [10,14,15]. Diagnostic differentiation between these infections remains challenging, as clinical signs are often nonspecific and direct detection methods show limited sensitivity in chronic cases [1,2,3,4].

Serological methods are therefore widely applied for the surveillance of equine trypanosomosis, particularly the complement fixation test (CFT) and the immunofluorescence antibody test (IFAT), which are currently recommended by the World Organisation for Animal Health for the diagnosis of dourine [16,17,18]. However, while suitable for large-scale screening, these methods cannot reliably distinguish between active and past infections and may be affected by cross-reactivity with other trypanosome species [19].

Although dourine has been reported historically in Kazakhstan, comprehensive and up-to-date nationwide data on its current epidemiological status remain limited. The lack of continuous surveillance hampers accurate risk assessment and the identification of potential epizootic foci, particularly in regions characterized by intensive horse breeding and animal movement.

The aim of this study was to conduct a nationwide serological survey of equine trypanosomosis across all administrative regions of Kazakhstan using complement fixation testing. By analysing the geographic distribution of seropositive animals, this work provides baseline data on antibody reactivity to equine trypanosomes and supports future surveillance and diagnostic refinement efforts.

## 2. Materials and Methods

### 2.1. Study Area and Animals

Kazakhstan is the largest landlocked country in the world, with a total land border length of approximately 13,200 km. The country’s landscape is predominantly composed of deserts (44%) and semi-deserts (14%), while steppes cover about 26% and forests make up around 5.5% of the national territory. These diverse ecological zones influence the distribution and density of equine populations and potential vectors.

**Figure 1:**
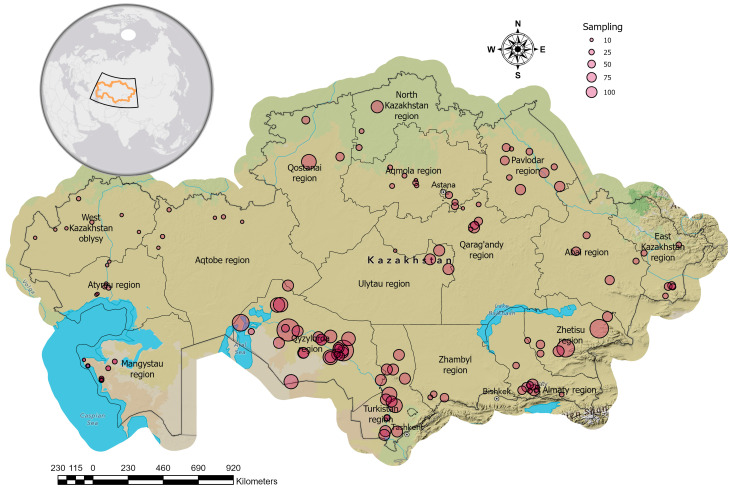
Study area: Map highlighting the sampling locations across Kazakhstan. Sampling locations are indicated by pink circles, with different sizes corresponding to the number of samples collected at each location.

This study was conducted between 2023 and 2025 and aimed to investigate the seroprevalence of dourine in equestrian farms across Kazakhstan. Serum samples were collected from various administrative regions representing all 17 regions of the country. The sampling sites were selected based on horse population density and accessibility. A detailed map of the sampling locations is provided in Figure 1.

### 2.2. Sample Collection

Whole blood samples were collected from the horses by jugular venipuncture into 10 mL siliconised plain and EDTA coated vacutainer tubes. One millilitre of EDTA-blood was aliquoted into cryovials and transferred into liquid nitrogen on the day of sampling. Blood samples collected within the silicon coated tubes were left to clot overnight.

The next day, one millilitre of serum was aspirated and placed in cryovials, which were then transferred to liquid nitrogen. The samples were kept in liquid nitrogen or frozen at −20 °C until tested by CFT/PCR. Horses were sampled during routine veterinary surveillance activities at participating farms, with animals selected in an unbiased manner to ensure broad representation of age, sex, and management conditions within each herd. The sampled population primarily consisted of working and breeding horses typical for regional husbandry systems, and most animals showed no overt clinical signs of dourine at the time of sampling.

### 2.3. CFT Protocol

The complement fixation test (CFT) was performed in accordance with the WOAH Terrestrial Manual using a commercially available diagnostic kit produced by the Kazakh Scientific Research Veterinary Institute. Serum samples were initially tested at a dilution of 1:5, and the degree of haemolysis inhibition (HI) was visually assessed. Reactions showing ≥50% HI were considered positive. Positive sera were further titrated by serial twofold dilutions to determine endpoint titres, which were calculated according to WOAH-recommended criteria. Based on this approach, the diagnostic cut-off for positivity corresponded to a titre value of 5, as defined by the WOAH Terrestrial Manual [20].

### 2.4. Molecular Detection by Real-Time PCR

PCR analysis was performed on all 287 CFT-seropositive samples using commercial TaqMan probe-based real-time PCR kits for the detection of *T. equiperdum* and *T. evansi* (BioinGentech Ltd., Concepción, Chile), according to the manufacturer’s instructions. According to the manufacturer, the assays target the plsX gene for *T. equiperdum* and the variant surface glycoprotein (VSG) gene for *T. evansi*.

### 2.5. Field Data Collection and Mapping Methods

Field data were collected using Survey123 for ArcGIS (version 4.15), a mobile geographic information system (GIS) application optimized for field-based data entry. Spatial data analysis, integration, and cartographic visualization were performed using ArcGIS Pro 3.4 (Esri Inc., Redlands, CA, USA) [21]. Field observations from Survey123 were directly imported into the software and integrated with auxiliary datasets, including topographic layers and administrative boundaries.

Cartographic outputs were designed according to principles of thematic clarity and visual hierarchy to ensure consistent interpretation across all map products [22]. Dynamic map elements such as scale bars and legends were standardized across layouts. Final maps were exported in high resolution (1000 dpi) using the WGS 1984 UTM coordinate system for publication.

### 2.6. Ethics Statement

The study protocol was reviewed and approved by the Biological Ethic Committee of the Kazakh Scientific Research Veterinary Institute (Protocol No. 1 from 14 July 2023).

### 2.7. Statistical Analysis

Descriptive statistics were used to estimate overall and regional seroprevalence, expressed as percentages with corresponding 95% confidence intervals (CIs). Differences in seroprevalence between geographic regions and between sexes were assessed using the Chi-square (χ^2^) test, with statistical significance set at *p* < 0.05.

The strength of associations between categorical variables (e.g., region and seropositivity, sex and seropositivity) was quantified using Cramér’s V coefficient and interpreted as negligible (<0.1), weak (0.1–0.3), moderate (0.3–0.5), or strong (>0.5). To account for potential overdispersion and unequal sample sizes across regions, a binomial generalized linear mixed-effects model (GLMM) with region as a random intercept was applied, allowing estimation of overall seroprevalence while quantifying the proportion of variability attributable to regional clustering. For sex-based comparisons, the Phi coefficient was additionally calculated to provide a standardized measure of association suitable for binary categorical variables.

Confidence intervals were calculated using the Wilson score method without continuity correction. Statistical analyses were conducted using IBM SPSS Statistics version 27 (IBM Corp., Armonk, NY, USA) and R software (version 4.4.0). Data preprocessing and visualization were performed using the *tidyverse* and *ggplot2* packages, with multi-panel figures assembled using *patchwork* and *ggpubr*. Spatial analyses and mapping were conducted in ArcGIS Pro 3.4 (ESRI, Redlands, CA, USA) [22]. All analyses were carried out using reproducible scripted workflows.

## 3. Results

**Figure 2:**
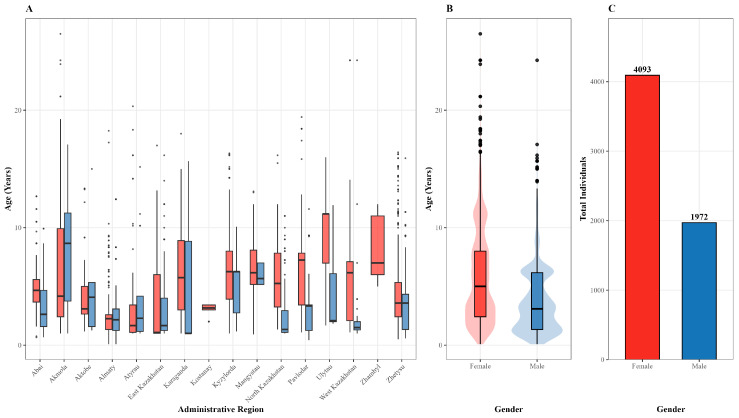
Age and sex structure of the examined horse population in Kazakhstan: (**A**) regional age distribution by sex; (**B**) national age distribution by sex; (**C**) total number of examined animals by sex.

As part of the seroepidemiological monitoring of trypanosomosis in horses, a total of 6065 equine serum samples were collected from all 17 administrative regions of Kazakhstan. The demographic structure of the examined population, including age and sex distribution by region, is presented in Figure 2.

**Figure 3:**
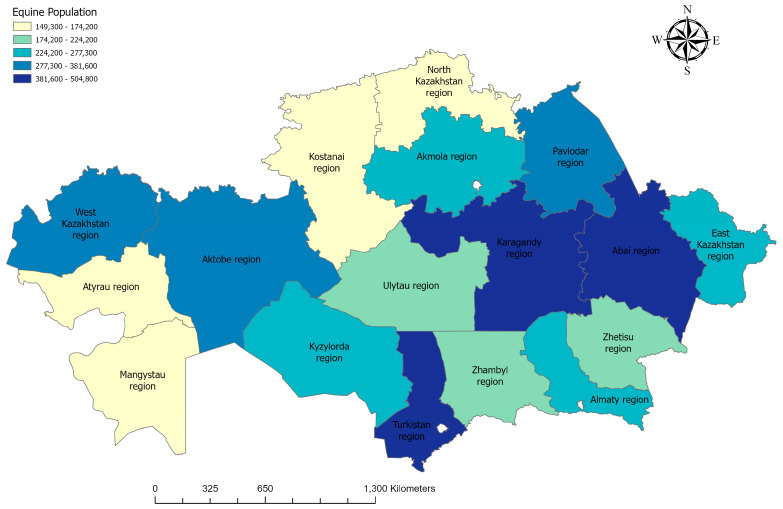
Map of the equine population in Kazakhstan. Statistical data for 2025 were obtained from the Bureau of National Statistics (https://stat.gov.kz) (accessed on 12 December 2025) [23].

Analysis of horse population dynamics, based on data as of March 1 for the period 2021–2025, shows a steady upward trend. The lowest recorded number of horses was in 2021, with 3.49 million head, whereas in 2025 the population reached its peak at 4.80 million head. National horse population dynamics for the study period are shown in Figure 3 [23].

**Figure 4:**
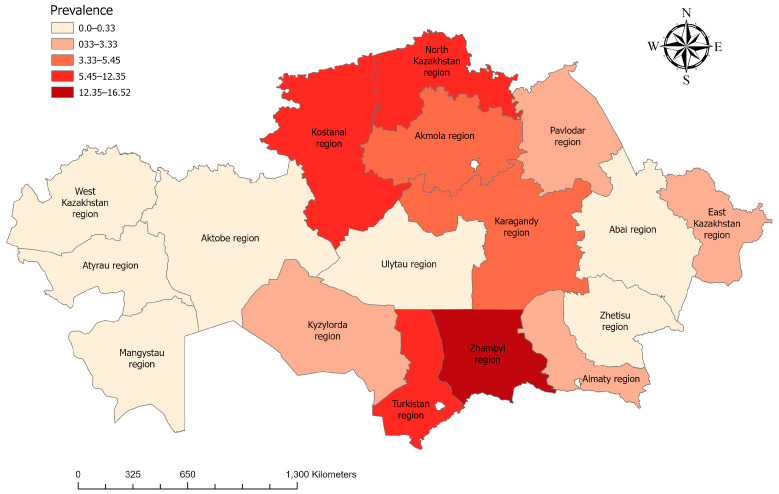
Epidemiological mapping of horse trypanosomoses. Each region is distinguished by a color gradation, which depends on the seroprevalences for horse trypanosomes. The ranges of the positive ratio for each color gradation are indicated in the figure.

Serological testing using the complement fixation test (CFT) identified 287 samples as positive, resulting in an overall seropositivity rate of 4.73%. Seropositive animals were detected in multiple regions, demonstrating a wide geographic distribution of antibody reactivity to *Trypanozoon* parasites (Figure 4).

**Figure 5:**
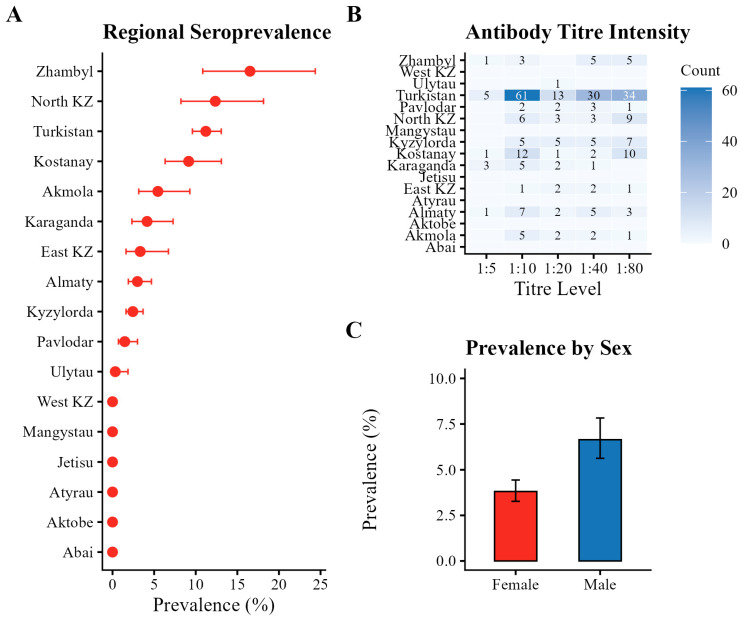
Epidemiological characteristics of *Trypanosoma equiperdum* infection in the examined horse population of Kazakhstan: (**A**) regional seroprevalence with CI, illustrating spatial heterogeneity in infection rates; (**B**) heatmap of antibody titre distribution across regions; (**C**) Comparison of seroprevalence between sexes.

Regional seroprevalence varied substantially across Kazakhstan (Figure 5A). The highest proportions of seropositive animals were recorded in Zhambyl Region (16.52%; 95% CI: 10.84–24.37), North Kazakhstan Region (12.35%; 95% CI: 8.22–18.15), and Turkistan Region (11.21%; 95% CI: 9.60–13.07). Moderate levels were observed in Kostanay (9.15%), Akmola (5.45%), and Karaganda (4.15%) Regions. Lower seropositivity rates ranging from 1.47% to 3.33% were identified in Pavlodar, East Kazakhstan, and Almaty Regions. No seropositive animals were detected in Mangystau, Atyrau, West Kazakhstan, Aktobe, Abai, and Jetisu Regions.

Pearson’s Chi-square test confirmed statistically significant differences in seropositivity between regions (χ^2^ = 290; df = 16; *p* < 0.001). Cramér’s V coefficient was 0.216, indicating a weak-to-moderate association between geographic region and antibody detection.

To account for unequal sample sizes and potential clustering, a binomial generalized linear mixed-effects model (GLMM) with region as a random intercept was fitted. The estimated variance of the regional random effect was 3.43 (SD = 1.85), corresponding to an intraclass correlation coefficient (ICC) of approximately 0.51. This indicates that more than half of the observed variability in seropositivity could be attributed to differences between regions.

Antibody titres among seropositive animals varied substantially (Table 1; Figure 5B). The most frequently observed titres were 1:10 and 1:80. Titres ≥ 1:40 were detected in 43.9% of seropositive samples. These variations likely reflect differences in the intensity of humoral immune responses or cumulative antigenic exposure. However, in the absence of longitudinal follow-up data, antibody titres cannot be used to infer the timing, duration, or activity of infection.

Seropositivity differed between sexes (Table 2; Figure 5C). Among male horses (*n* = 1972), 131 were seropositive (6.64%; 95% CI: 5.63–7.83%), whereas among females (*n* = 4093), 156 were seropositive (3.81%; 95% CI: 3.27–4.44%).

The difference was statistically significant (χ^2^ = 23.05; df = 1; *p* < 0.001). However, the Phi coefficient was 0.062, indicating a very weak association between sex and antibody detection.

A comprehensive visualization of regional seroprevalence, antibody titre distribution, and sex-based differences is provided in Figure 5.

PCR analysis was performed on 287 seropositive samples. The *T. equiperdum* plsX gene was not detected in any of the tested samples. One sample from the Almaty Region tested positive for the *T. evansi* variant surface glycoprotein gene.

These molecular findings indicate that CFT seropositivity in this study was not accompanied by molecular confirmation of *T. equiperdum* infection.

## 4. Discussion

Equine trypanosomosis remains an important veterinary concern in regions where horses play a significant economic and cultural role. Although dourine has been historically reported in several countries, including Kazakhstan, official notifications of confirmed *Trypanosoma equiperdum* or *Trypanosoma evansi* infections have not been submitted by Kazakhstan to the World Organisation for Animal Health over the past two decades [19,24,25]. In this context, the present study provides updated nationwide data on serological reactivity to equine trypanosomes across all administrative regions of the country.

Serological surveillance plays a central role in the monitoring of equine trypanosomosis, particularly in large-scale epidemiological investigations. According to WOAH recommendations, tests such as IFAT, ELISA, CFT, and CATT/*T. evansi* are routinely applied for the diagnosis of dourine and related trypanosome infections [17,26]. Among these, the complement fixation test remains widely used due to its practicality and suitability for screening large populations. However, it is well established that CFT detects antibodies against members of the *Trypanozoon* subgenus and does not reliably distinguish between *T. equiperdum* and *T. evansi* [24,27]. Furthermore, seropositivity reflects exposure to trypanosome antigens and does not equate to confirmed infection or clinical disease.

In the present study, 4.73% of examined horses were seropositive by CFT, with significant regional heterogeneity. Importantly, PCR testing of 287 seropositive samples did not detect the *T. equiperdum* plsX gene, while one sample was positive for *T. evansi*. The absence of molecular confirmation of *T. equiperdum* indicates that the detected serological reactivity cannot be interpreted as confirmed dourine infection. Instead, these findings suggest prior or ongoing exposure to equine trypanosomes, with possible cross-reactivity between closely related *Trypanozoon* species.

A central finding of the present study is the discordance between serological and molecular results. Although 287 horses tested seropositive by CFT, PCR analysis did not confirm the presence of *T. equiperdum*, and only one sample was positive for *T. evansi*. This discrepancy warrants careful interpretation.

Several explanations may account for this pattern. First, equine trypanosomosis, particularly dourine, is characterized by chronic infection with low or intermittent parasitemia. Parasites may localize predominantly in tissues rather than circulating in peripheral blood, thereby reducing the sensitivity of blood-based molecular detection. Consequently, PCR-negative results do not necessarily exclude prior infection. Furthermore, the absence of PCR detection in peripheral blood samples does not exclude the presence of tissue-localized parasites during chronic infections, and molecular negativity should not be interpreted as confirmation of active infection.

Second, antibodies may persist for extended periods following exposure. Serological positivity therefore reflects immunological memory rather than active parasitemia. In endemic or previously exposed populations, long-term antibody persistence can lead to continued seroreactivity even after parasite clearance.

Third, cross-reactivity between closely related *Trypanozoon* species must be considered. The detection of *T. evansi* in one seropositive sample supports the possibility that CFT reactivity may reflect exposure to different members of the *Trypanozoon* subgenus rather than species-specific infection. Given the antigenic similarity between *T. equiperdum* and *T. evansi*, serological differentiation remains challenging.

Fourth, the complement fixation test has recognized diagnostic limitations. Although recommended by WOAH for dourine surveillance, CFT does not provide species-level resolution and may yield false-positive or nonspecific reactions depending on antigen composition and serum characteristics. The absence of internationally validated performance characteristics for the locally produced kit further emphasizes the need for cautious interpretation.

These considerations have important implications for WOAH-aligned surveillance strategies. Serological screening remains essential for large-scale monitoring; however, species attribution and confirmation of active dourine require molecular validation and the development of more specific diagnostic tools. Integrating serological and molecular approaches will be critical for accurately defining the epidemiological status of equine trypanosomosis in Kazakhstan.

Similar diagnostic challenges have been described in other endemic regions, including Mongolia, where differentiation between *T. equiperdum* and *T. evansi* has proven difficult using conventional serological methods [27]. Cross-reactive or nonspecific reactions in CFT may arise due to antigen composition, anticomplementary serum activity, or the use of rat-derived parasite strains [9,20,28]. These limitations underscore the need for molecular confirmation and the development of recombinant, species-specific antigens to improve diagnostic precision.

The observed regional heterogeneity in seropositivity likely reflects differences in herd management practices, animal movement, population density, and local epidemiological contexts. However, as this study was not designed to formally evaluate specific risk factors, causal interpretations should be made cautiously. The regional clustering detected in mixed-effects modelling highlights the importance of geographically targeted surveillance strategies.

Although region-level effects were addressed using a mixed-effects modelling approach, a fully multivariate analysis incorporating individual-level covariates such as sex could not be performed due to the structure of the available data. Individual animal characteristics and serological results were not consistently linked across all regions, which limited the inclusion of multiple predictors within a single model. Future surveillance studies integrating harmonized individual-level datasets will enable more comprehensive multivariate risk factor analyses.

Several limitations must be acknowledged. First, a limitation relates to the diagnostic validation of the locally produced CFT kit. Although the assay is implemented in national surveillance programs and follows WOAH methodological principles, its performance characteristics have not been evaluated through independent international validation studies. Sensitivity, specificity, and cross-reactivity parameters have therefore not been formally established using standardized reference panels. As a consequence, species-level attribution based solely on CFT results remains uncertain. Seropositivity should be interpreted as evidence of antibody reactivity to *Trypanozoon* antigens rather than definitive identification of *T. equiperdum*. This diagnostic uncertainty has direct implications for surveillance accuracy, particularly in distinguishing between exposure to different *Trypanozoon* species and confirming active dourine.

Future studies incorporating internationally validated assays and harmonized molecular confirmation protocols will be essential for improving diagnostic precision and ensuring reliable epidemiological classification.

Second, molecular testing was limited to selected targets and may not detect all circulating strains. Third, individual-level variables were not uniformly linked to serological results across all regions, limiting multivariate risk analysis. Also, the horses included in this survey represented a heterogeneous equine population typical of regional husbandry systems in Kazakhstan. Most animals were clinically asymptomatic at the time of sampling, and systematic data on individual animal movement or detailed clinical history were not available. These characteristics are consistent with large-scale surveillance-based studies and limit the ability to correlate serological findings with clinical status or disease progression.

Importantly, exposure, infection, and clinical disease represent distinct epidemiological states. Seropositivity indicates immunological evidence of contact with trypanosome antigens but does not confirm active parasitemia or clinical dourine. Most animals included in this survey were clinically asymptomatic at the time of sampling, and detailed clinical histories were not systematically available. These characteristics are consistent with large-scale surveillance studies but limit interpretation regarding disease burden.

Taken together, the present findings provide the first nationwide overview of serological reactivity to equine trypanosomes in Kazakhstan. While the results demonstrate geographically heterogeneous antibody detection, species-level attribution and confirmation of active dourine require further molecular investigation. Strengthening integrated serological and molecular surveillance, harmonizing diagnostic standards with international recommendations, and developing more specific assays will be essential for accurately defining the epidemiological status of equine trypanosomosis in the region [29].

## 5. Conclusions

This study presents the first nationwide serological survey of equine trypanosomosis in Kazakhstan, covering all 17 administrative regions of the country. A total seropositivity rate of 4.73% was detected using the complement fixation test, with significant regional heterogeneity.

The findings demonstrate geographically distributed serological reactivity to *Trypanozoon* parasites among equine populations. However, the absence of molecular confirmation of *Trypanosoma equiperdum* indicates that these results should be interpreted as evidence of exposure rather than confirmed dourine infection.

The study provides baseline data for future integrated surveillance efforts combining serological and molecular approaches. Continued refinement of diagnostic tools and harmonization with international standards will be essential for accurately defining the epidemiological status of equine trypanosomosis in Kazakhstan.

## Figures and Tables

**Table 1:** Seropositive samples in the studied regions divided by antibody titres.

Regions of Kazakhstan	Antibody Titres						Total
	1:5	1:10	1:20	1:40	1:60	1:80	
Turkistan	5	61	13	30	0	34	143
Almaty	1	7	2	5	0	3	18
Zhambyl	1	3	0	5	0	5	19
Kyzylorda	0	5	5	5	0	7	22
Ulytau	0	0	1	0	0	0	1
North Kazakhstan	0	6	3	3	0	9	21
Kostanay	1	12	1	2	0	10	26
Karaganda	3	5	2	1	0	0	11
Akmola	0	5	2	2	0	1	12
Mangystau	0	0	0	0	0	0	0
Atyrau	0	0	0	0	0	0	0
West Kazakhstan	0	0	0	0	0	0	0
Aktobe	0	0	0	0	0	0	0
East Kazakhstan	0	1	2	2	0	1	7
Pavlodar	0	2	2	3	0	1	8
Abai	0	0	0	0	0	0	0
Jetisu	0	0	0	0	0	0	0
Total	11	108	33	58	0	68	287

**Table 2:** Sex-wise seroprevalence of equine trypanosome seroreactivity.

Sex	No. of Equines Examined (*n*)	No. Seropositive (*n*)	Serorevalence (%)	95% CI
Male	1972	131	6.64	5.63–7.83%
Female	4093	156	3.81	3.27–4.44%

Note: Sex-wise prevalence by Chi-square test: χ^2^ = 23.05, df = 1, *p* < 0.001.

## Data Availability

The original contributions presented in this study are included in the article. Further inquiries can be directed to the corresponding author.

## Abbreviations

The following abbreviations are used in this manuscript:

CFT	Complement fixation test
WOAH	World Organisation for Animal Health
PCR	Polymerase chain reaction
HI	Haemolysis inhibition
IFAT	Immunofluorescence antibody test
IHA	Indirect hemagglutination assay
ELISA	Enzyme-linked immunosorbent assay
rELISA	Recombinant ELISA
CI	Confidence interval
GIS	Geographic information system
KazSRVI	Kazakh Scientific Research Veterinary Institute
IC	Inconclusive serum
